# Global Combustion CO_2_ Accounting Neglects the Origin of Traded Fossil Fuels

**DOI:** 10.1021/acs.est.6c01630

**Published:** 2026-08-27

**Authors:** Binyuan Liu, Yuli Shan, Yuru Guan, Steven J. Davis, Ye Hang, Liang Jing, Xiande Ji, Wenqiang Wang, Klaus Hubacek

**Affiliations:** † Energy and Sustainability Research Institute Groningen, 3647 University of Groningen, Groningen 9747 AG, The Netherlands; ‡ School of Geography, Earth and Environmental Sciences (GEES), 1724 University of Birmingham, Birmingham B15 2TT, U.K.; § Department of Earth System Science, Stanford University, Stanford, California 94305, United States; ∥ Energy Sustainability Analysis, Technology Strategy and Planning Department, 36516 Saudi Aramco, Dhahran 31311, Saudi Arabia; ⊥ Institute of Blue and Green Development, 12589 Shandong University, Weihai 264209, China

**Keywords:** emission accounting, emission factors, fossil fuel trade, national inventory uncertainty, IPCC Tier 1, Nationally Determined Contributions

## Abstract

Combustion CO_2_ emissions are commonly estimated by multiplying fuel consumption by the corresponding emission factors. However, national inventories may apply domestic factors to imported fuels, overlooking differences in fuel properties across extraction origins and creating discrepancies in emission estimates. Here, we trace fossil fuels to their countries of extraction and assign emission factors specific to the extraction country and fuel type. Using 2020 data for 150 countries and regions, we compare this origin-traced estimate with a nonorigin-traced control using the same energy-consumption data but combustion-country emission factors. Globally, the control estimate is 339.2 Mt of CO_2_ lower than the origin-traced estimate, equivalent to 1.0% of the origin-traced total, with coal accounting for 89.9% of the discrepancy. Although crude oil had the largest energy-based trade volume among primary fuels, oil-related fuels contributed less because their emission factors varied less across countries, particularly for refined petroleum products. Such discrepancies can be particularly consequential for highly import-dependent countries. For example, the national-level discrepancy exceeds 4% in India, which is a major fossil-fuel importer. Our origin-tracing approach can improve the accuracy of combustion CO_2_ estimates by assigning emission factors that better reflect the fuels actually consumed. This provides more robust emission information for assessing progress toward Nationally Determined Contributions.

## Graphical abstract

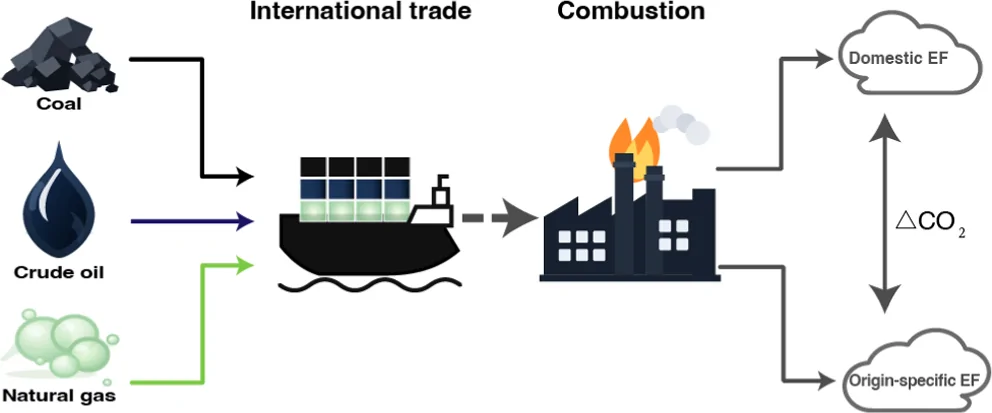

## 1 Introduction

Anthropogenic activities, particularly the extensive use of fossil fuels for economic production, are the leading drivers of global warming.^1^ Accurate greenhouse gas (GHG) emission estimates are critical for quantifying the contributions of human activities to climate change, informing targeted mitigation strategies, and assessing national efforts to reduce emissions.^2,3^ The Intergovernmental Panel on Climate Change (IPCC) recommends estimating energy-related emissions based on activity data and corresponding emission factors.^4^ While efforts have been made to improve the quality of activity data,^5,6^ variations in emission factors across fuel origins can still introduce additional uncertainty into emission estimates. This issue is reflected in generalized emission factor treatments. The IPCC Tier 1 method applies default emission factors to national activity data.^7^ Because the fuel origin is not explicitly distinguished in this treatment, it may not capture variation in emission factors among fuels of the same category that originate in different regions.

However, even within the same fuel category, properties may differ across regions. Based on IEA data, the relative difference between the highest and lowest country-level net calorific values, calculated separately for each of five coal categories (anthracite, coking coal, other bituminous coal, sub-bituminous coal, and lignite), ranged from 69% to 112%.^8^ For example, Liu et al. highlighted such regional differences, concluding that emission factors for Chinese coal are, on average, 40% lower than the IPCC defaults, while those for natural gas are 13% higher.^9^ Such variation can arise from origin-specific differences in fuel composition and energy content. For primary fuels, these differences are shaped by geological formation conditions.^10,11^ For secondary fuels, particularly refined petroleum products, composition is shaped by refining processes designed to meet product-specific performance requirements.^12–14^ These origin-related variations are captured in the carbon content, net calorific value, and oxidation factor used to derive CO_2_ emission factors. They can therefore affect the accuracy of national emission inventories.

Related studies have shown that heterogeneity in fossil fuel sources and supply chains can affect carbon intensity estimates, particularly in life-cycle analyses of crude oil production,^15^ refining,^16^ and crude oil trading.^17^ Previous work has also traced global CO_2_ emissions along the supply chain, from fossil fuel extraction to production-based emissions and final consumption.^18^ However, these studies mainly focus on life-cycle carbon intensity or the attribution of CO_2_ emissions along supply chains, rather than on how the origin of fuel extraction affects emission factor assignment and, in turn, the accuracy of national CO_2_ inventories.

In national inventories, by contrast, generalized emission factor treatments remain common. Tier 1 methods provide a pragmatic approach, particularly in data-scarce contexts, and many global emission databases likewise rely on this generalized approach without accounting for differences in fuel origin.^19–21^ This limitation becomes particularly pronounced for countries heavily reliant on imported fossil fuels because imports often combine supplies from multiple source countries with different fuel properties. National greenhouse gas inventories, however, typically do not distinguish the multiple origins within an imported fuel category, making it difficult to assign an emission factor that accurately reflects the underlying origin mix. The challenge is further compounded by the complexity of international trade, which can obscure the original source of fossil fuels before final use.^18^

To address this gap, we propose an approach that uses global fossil fuel trade data to trace imported fuels back to their countries of extraction and assigns origin-specific emission factors accordingly. Building on a supply chain perspective, our approach brings fuel origin information into national combustion CO_2_ inventories, enabling us to assess how the origin of imported fossil fuels affects emission factor assignment and, in turn, national emission estimates. Assigning origin-specific emission factors can improve the consistency between the selected emission factors and fuel properties shaped by geological and processing conditions. This provides a practical route to make emission factors for imported fuels more representative when origin-specific fuel information is available. Our analysis focuses on fossil fuel combustion CO_2_ and covers 150 countries and regions in 2020, using 39 IEA fuel products selected for origin tracing (14 coal and coal products; 21 crude oil and petroleum-related products; natural gas; and three other solid fuels: peat, peat products, and oil shale and oil sands). Emission factors are assigned separately for each origin and fuel-type combination. To evaluate the effect of fuel-origin tracing, we compare our results with a nonorigin-traced control estimate that uses the same energy consumption data but assigns emission factors according to the country where fuels are combusted. Although the present study focuses on 2020 as a demonstration year, the approach can, in principle, be extended to examine how changes in trade patterns affect the origin mix and emission factors of imported fuels over time.

## 2 Materials and Methods

### 2.1 Modeling Global Fossil-Fuel Trade Patterns

#### 2.1.1 Data Sources

To assign emission factors that reflect the extraction origin of fuels ultimately combusted in each country, we first constructed a global fossil-fuel trade matrix by combining country-level physical energy statistics from the International Energy Agency (IEA) with bilateral trade shares from UN Comtrade. The resulting matrix provides the origin shares needed to allocate emission factors to the fuels consumed in each country.

We extract the country-specific data on energy supply (production, imports, exports, and stock changes) and energy use (including transformation inputs) from the IEA’s World Energy Statistics.^22^ This data set covers 38 types of fossil fuels, reported in thousand tonnes. Additionally, we use the IEA’s World Natural Gas Statistics for natural gas data,^23^ expressed in million cubic meters. Together, these data cover 150 countries and regions for 39 types of fossil fuel in 2020. For natural gas, bilateral trade flows were obtained directly from the IEA’s dedicated natural-gas trade data. For solid and liquid fuel products, the IEA provides aggregate trade totals but not bilateral trade flow. We therefore use the UN Comtrade database^24^ to derive bilateral trade shares, while retaining IEA totals as the benchmark physical trade volumes for each country and fuel type. Since UN Comtrade is used to estimate relative bilateral shares rather than final absolute trade volumes, differences in reporting units between the two data sets do not affect the absolute trade volumes in the final harmonized trade matrix. For each country and fuel type, bilateral trade shares are calculated from comparable quantity records in UN Comtrade, normalized across trading partners, and then applied to the corresponding IEA trade totals. In this way, the final bilateral trade matrix preserves the physical totals reported by the IEA while using UN Comtrade to approximate the partner structure of fossil fuel trade flows. Additionally, the Global Trade Analysis Project (GTAP), which provides bilateral trade flows for five types of fossil fuel from 141 countries/regions in 2017,^25^ is used as Supporting Data in cases where the UN Comtrade database lacks records.

Emission factors are derived from the multiplication of several critical variables: the net calorific value representing the amount of energy released per unit mass of solid and liquid fuels, or per unit volume of gaseous fuels; the carbon content, denoting the amount of carbon per unit of energy; and the oxidation factor, indicating the proportion of carbon oxidized during combustion.^4^ We obtained the country-specific net calorific values for coal, oil, and their products from the IEA’s World Conversion Factors (WCONV) database.^8^ WCONV reports gross calorific values by flow for natural gas, which we converted to net calorific values using a factor of 0.9 before constructing natural-gas emission factors. Carbon content values by fuel type were collected from the IPCC Emission Factor Database.^26^ However, only a limited number of countries provide specific carbon content data. In our analysis, we distinguished carbon content values for certain fossil fuel products originating from China, the US, Japan, and several EU countries. For most other countries, we used the default values due to the widespread lack of country-specific data. Similarly, country-specific data for oxidation factors are limited. Therefore, we assumed complete combustion for all fuels, setting the oxidation factor to 1.

#### 2.1.2 Processing of Trade Data

The UN Comtrade database provides detailed country-level trade data in physical units. These data are reported by individual countries or regions, which may follow different standards and methodologies for data collection and measurement. The resulting records can therefore be inconsistent across countries.^27,28^ To address the issue of poor data comparability, we use UN Comtrade only to derive bilateral export shares, while IEA data provide the corresponding national export totals. Before calculating the shares, we subtracted re-exports from exports using the re-export records available in UN Comtrade. Using the countries and energy types identified in the IEA database, we then extracted export shares by destination country for each fossil fuel type and used these shares to disaggregate the national total export data from the IEA (see eq 1).

1 Exportsr→j,i=IEAexpr,i×(UNr→j,i∑UNr,i)

where ${Exports}^{r\to j,i}$ represents the export of energy type *i* from country *r* to country *j*. IEA_exp_^*r*,*i*^ refers to the total exports of energy type *i* from country *r*, based on the data from IEA. ${UN}^{r\to j,i}$ means the export of energy type *i* from country *r* to country *j*, while ∑UN^*r*,*i*^ denotes the total export of energy type *i* from country *r*, as reported in the UN Comtrade database.

Although UN Comtrade also reports import data, mirror trade records often differ because of differences in statistical criteria and reporting practices.^28^ We therefore construct the import matrix from exporter-reported bilateral flows and scale it to the corresponding IEA import totals (see eq 2).

2 $${Imports}^{r\to j,i}={IEA}_{imp}^{j,i}\times \left(\frac{{Exports}^{r\to j,i}}{\sum {Exports}^{j,i}}\right)$$

where ${Imports}^{r\to j,i}$ indicates the import of energy type *i* from country *r* to country *j*. IEA_imp_^*j*,*i*^ refers to country *j*’s total imports of energy type *i*, based on the data from IEA. ∑Exports^*j*,*i*^ represents the total exports of energy type *i* to country *j*.

In the matching process, we observed that the energy product classifications in the IEA database and the UN Comtrade database are not fully aligned. Specifically, the UN Comtrade database organizes its commodity classification according to multiple international standards; we used the Harmonized System (HS) classification, as it provides detailed insight into trade flows of specific energy products.^28,29^ In particular, fossil energy is categorized into 15 major classes (HS code: 2701 to 2715), each of which includes several subdivisions, totaling 63 subcategories. In comparison, the IEA’s classification of energy types is slightly less extensive, with 43 out of 68 energy product categories related to fossil fuels. Of these 43 fossil-related IEA products, we selected 39 disaggregated products for origin tracing. The remaining four are aggregate categories that overlap with more disaggregated products (e.g., hard coal, which overlaps with anthracite, coking coal, and bituminous coal) or lubricants, which are outside the combustion-accounting scope. We therefore did not retain these products in the product-level mapping. We developed a product-level crosswalk between the two classifications; the detailed results are provided in the Supporting Information, Data S2. While the overall compatibility of energy categories between the two databases is high, some mismatches persist. For example, both the IEA and UN Comtrade record coal exports from Peru; however, the IEA classifies it as bituminous coal, while UN Comtrade lists it as anthracite. Such inconsistencies account for 0.5% of the total trade volume, which we addressed by retaining the export ratios from UN Comtrade and aligning the classifications with IEA data.

Additionally, in the UN Comtrade database, quantity and net weight data are occasionally missing.^28^ These omissions account for the majority of the unmatched cases. We bridge these gaps by applying alternative ratio estimates according to the following principles:

Broader category ratios: in cases where specific subcategory export data are missing, we use the export ratios of broader categories. For example, within HS code 2711, which covers petroleum gases and other gaseous hydrocarbons, export data from Qatar only provide information at the broader category level, without distinguishing among specific petroleum-gas subcategories, such as propane (271112) and butanes (271113). Thus, the export ratio of HS 2711 is used uniformly across all of its subcategories.

Adjacent year ratios: where broader category ratios are unavailable, we apply ratios from adjacent years. This approach is particularly relevant for Iran, for which 2021 provides a more complete data set compared to 2020.

GTAP ratios: if no export ratio could be obtained using the above approaches, we used the data from the GTAP, which provides bilateral trade flows for 141 countries/regions in 2017. We consider this substitution to be justifiable. Although the relevant GTAP bilateral trade flows are not directly reported in physical volumes but are derived from monetary trade flows, the internally consistent modeling framework underpinning the database supports its use as an appropriate alternative in these cases.^25^

For countries lacking any available export ratio, we apply the global average as a substitute. Given these countries’ minimal export volumes, this substitution has a negligible effect on the overall results.

### 2.2 Emission Accounting

We estimated fossil-fuel-related CO_2_ emissions based on a carbon mass balance approach

3 Emission=activitydata×EF

where activity data represent the amount of fossil fuels consumed within the territorial boundary, while EF refers to the emission factors for each fuel type in each country.

In general, energy consumption can be calculated in one of two ways: as “apparent energy consumption (AC)” from the energy supply perspective, or as “final and input/output consumption” from the energy usage perspective.^30^ The AC approach, based on domestic fuel production, international trade, and stock changes^9,31^ (see eq 4), facilitates tracing fossil fuels back to their origin countries.

4 AC=DomesticProduction+Imports−Exports±StockChange

In eq 4, a positive stock change represents stock drawdown and is added to apparent consumption, whereas a negative stock change represents stock build-up and is subtracted from apparent consumption. To prevent double counting, this approach includes only three primary fuel types (raw coal, crude oil, and natural gas), while excluding secondary fuels such as gasoline and electricity, which are derived from these primary sources.^32^ This simplification, however, overlooks the extensive trade of secondary fuels, particularly oil products (e.g., a country refining crude oil into gasoline and exporting it, where emissions from the exported oil products are still attributed to the refining country). In contrast, the “final and input/output consumption” approach focuses on sector-specific energy use within a country,^32^ allowing for emission accounting at a microlevel. While this enables more granular and accurate assessments, it complicates the tracking of fossil fuel origins, particularly in cases involving complex international supply chains.

In this study, we use a hybrid approach that combines the advantages of both methods to account for production-based emissions at the national level. The approach retains the ability to trace traded fossil fuels back to the countries where they were originally extracted and to apply the corresponding origin-specific emission factors. At the same time, it distinguishes primary fuels from secondary fuels by identifying primary energy inputs used in transformation processes and accounting for emissions from secondary fuels when they are ultimately combusted. Specifically, we first quantify apparent consumption for primary energy (AC_pri_), primary energy input for transformation into secondary energy (FF_trans_), and apparent consumption for secondary energy (AC_sec_). The total energy consumption of fossil fuel (*E*_total_) can be expressed in eq 5

5 $$E_{total}={AC}_{pri}-{FF}_{trans}+{AC}_{\sec}$$

Applying eq 4 to primary fuels gives

6 $${AC}_{pri}^{r,i}={Prod}_{pri}^{r,i}+\sum_{s}{Imports}_{pri}^{s\to r,i}-{Exports}_{pri}^{r,i}\pm {Stock\;Change}_{pri}^{r,i}$$

In eq 6, Prod_pri_^*r*,*i*^ is domestic production of primary energy *i* in country *r*. $\sum_{s}{Imports}_{pri}^{s\to r,i}$ includes imports of primary energy *i* from all other regions *s* to country *r*, which is derived from the bilateral trade matrix constructed above. Exports_pri_^*r*,*i*^ refers to the total exports of primary energy *i* in country *r*. Stock Change_pri_^*r*,*i*^ indicates the stock change of primary energy *i* in country *r*.

The transformation process converts primary forms of energy into secondary forms,^33^ such as gasoline from crude oil or electricity and heat from coal. Transformation input data can be obtained from the IEA database. We differentiate transformation processes based on whether the conversion is achieved through combustion. For example, the conversion into coal products and oil products differs from the production of heat and electricity generation. The latter requires combustion and leads to CO_2_ emissions in the country where the transformation takes place. In contrast, the “actual emissions” from coal and oil products occur in the country where these converted products are ultimately burned. Therefore, we exclude coal and crude oil used for conversion into coal and oil products and instead account for the emissions arising from the combustion of coal and oil products. In this study, FF_trans_ specifically refers to the transformation processes that produce coal and oil products, excluding processes like electricity or heat generation.

We further differentiate FF_trans_ by origin because imported and domestically produced inputs are assigned different emission factors. Specifically, we can infer the imported energy ( ${FF}_{trans\text{\_}im}$ ) and domestic energy ( ${FF}_{trans\text{\_}dom}$ ) used in the transformation process by examining the proportion of imported energy in the total supply of primary energy *i* in country *r* (*R*_pri,im_^*r*,*i*^) following an approach previously outlined for tracing energy imports.^18^

It is also important to note that countries may utilize stock reserves to support exports or transformation processes. When stock reserves are depleted, we assume all these stocks come from previous domestic production. Conversely, when stock reserves increase, we further differentiate where these increased stocks come from. To account for this, the equation of *R*_pri,im_^*r*,*i*^ is calculated as follows, where ${\left|{Stock\;Change}_{pri}^{r,i}\right|}_{decrease}$ refers to the stock change that is consumed.

7 $$R_{pri,im}^{r,i}=\left({Imports}_{pri}^{r,i}\right)/\left({Prod}_{pri}^{r,i}+{Imports}_{pri}^{r,i}-{Exports}_{pri}^{r,i}+{\left|{Stock\;Change}_{pri}^{r,i}\right|}_{decrease}\right)$$

For each fossil fuel used in the transformation process (FF_trans_) within each country, the portion derived from imports ( ${FF}_{trans\text{\_}im}$ ) and the portion sourced domestically ( ${FF}_{trans\text{\_}dom}$ ) are expressed as follows

8 $${FF}_{trans\text{\_}im}^{s\to r,i}={FF}_{trans}^{r,i}\times R_{pri,im}^{r,i}\times R^{s\to r,i}$$

9 $${FF}_{trans\text{\_}dom}^{r,i}={FF}_{trans}^{r,i}\times \left(1-R_{pri,im}^{r,i}\right)$$

where ${FF}_{trans\text{\_}im}^{s\to r,i}$ and ${FF}_{trans\text{\_}dom}^{r,i}$ indicate the portions of primary energy *i* in country *r* used in the transformation process, derived from imports from country *s* and sourced domestically, respectively. $R^{s\to r,i}$ refers to the share of energy *i* imported from country *s* to country *r* ( $\sum_{s}R^{s\to r,i}=1$ ), as obtained from eq 2

For primary energy *i* in country *r* that has experienced a stock change increase ( ${\left|{Stock\;Change}_{pri}^{r,i}\right|}_{increase}$ ), we differentiate the portion derived from imports and the portion sourced domestically as follows

10 $${Stock\;Change}_{pri,increase}^{s\to r,i}={\left|{Stock\;Change}_{pri}^{r,i}\right|}_{increase}\times R_{pri,im}^{r,i}\times R^{s\to r,i}$$

11 $${Stock\;Change}_{pri,increase}^{dom,r,i}={\left|{Stock\;Change}_{pri}^{r,i}\right|}_{increase}\times \left({1-R}_{pri,im}^{r,i}\right)$$

where ${Stock\;Change}_{pri,increase}^{s\to r,i}$ refers to the portion of primary energy *i* stock increase in country *r* that comes from country *s*. Stock Change_pri,increase_^dom,*r*,*i*^ indicates the portion of primary energy *i* stock increase in country *r* that comes from domestic sources.

Apparent consumption of secondary fossil fuel *i* in country *r*, including coal and oil products, can be expressed as follows

12 $${AC}_{\sec}^{r,i}={Prod}_{\sec}^{r,i}+\sum_{s}{Imports}_{\sec}^{s\to r,i}-{Exports}_{\sec}^{r,i}\pm {Stock\;Change}_{\sec}^{r,i}$$

Stock changes in secondary fuels are treated in the same way as those in primary fuels. Stock drawdowns are attributed to previous domestic production, whereas stock build-ups are allocated between domestic and imported supply according to their respective shares in current secondary-fuel supply. The imported portion is further distributed across source countries using bilateral import shares. The resulting domestic and source-specific portions of secondary-fuel stock build-ups are denoted by Stock Change _sec,increase_^dom,*r*,*i*^ and ${Stock\;Change}_{\sec,increase}^{s\to r,i}$ , respectively.

Applying the origin-specific allocations described above to both primary and secondary fuels, eq 5 can be expanded as follows

13 $$E_{total}^{r}=\sum_{i}\left[\left({Prod}_{pri}^{r,i}+{\left|{Stock\;Change}_{pri}^{r,i}\right|}_{decrease}-{Exports}_{pri}^{r,i}-{Stock\;Change}_{pri,increase}^{dom,r,i}-{FF}_{trans\text{\_}dom}^{r,i}\right)+\sum_{s}\left({Imports}_{pri}^{s\to r,i}-{Stock\;Change}_{pri,increase}^{s\to r,i}-{FF}_{trans\text{\_}im}^{s\to r,i}\right)+\left({Prod}_{\sec}^{r,i}+{\left|{Stock\;Change}_{\sec}^{r,i}\right|}_{decrease}-{Exports}_{\sec}^{r,i}-{Stock\;Change}_{\sec,increase}^{dom,r,i}\right)+\sum_{s}\left({Imports}_{\sec}^{s\to r,i}-{Stock\;Change}_{\sec,increase}^{s\to r,i}\right)\right]$$

Therefore, energy-related emissions in country *r* can be expressed as follows

14 $${Emission}^{r}=\frac{44}{12}\times \sum_{i}\left[\left({Prod}_{pri}^{r,i}+{\left|{Stock\;Change}_{pri}^{r,i}\right|}_{decrease}-{Exports}_{pri}^{r,i}-{Stock\;Change}_{pri,increase}^{dom,r,i}-{FF}_{trans\text{\_}dom}^{r,i}\right)\times {NCV}_{r,i}\times {CC}_{r,i}\times {\text{OR}}_{r,i}+\sum_{s}\left({Imports}_{pri}^{s\to r,i}-{Stock\;Change}_{pri,increase}^{s\to r,i}-{FF}_{trans\text{\_}im}^{s\to r,i}\right)\times {NCV}_{s,i}\times {CC}_{s,i}\times {\text{OR}}_{s,i}+\left({Prod}_{\sec}^{r,i}+{\left|{Stock\;Change}_{\sec}^{r,i}\right|}_{decrease}-{Exports}_{\sec}^{r,i}-{Stock\;Change}_{\sec,increase}^{dom,r,i}\right){\times NCV}_{r,i}\times {CC}_{r,i}\times {\text{OR}}_{r,i}+\sum_{s}\left({Imports}_{\sec}^{s\to r,i}-{Stock\;Change}_{\sec,increase}^{s\to r,i}\right)\times {NCV}_{s,i}\times {CC}_{s,i}\times {\text{OR}}_{s,i}\right]$$

## 3 Results

### 3.1 Emission Factor Variability across Energy Types

Figure 1 illustrates emission factors plotted against log_10_-scaled energy export volumes for selected fuels and the distribution of the emission factors. Coal-based fuels, including lignite, subbituminous coal, bituminous coal, coking coal, and anthracite, exhibit a wider range of emission factors. For instance, lignite ranges from 0.49 tCO_2_/t in Greece to 1.96 tCO_2_/t in Kazakhstan. Several major coal exporters also deviate from the IPCC default values, as shown by the horizontal dashed lines in Figure 1. Indonesia has a relatively high emission factor for subbituminous coal, while Russia shows high values for both lignite and anthracite. In contrast, the emission factors of Australian coking coal and Indonesian bituminous coal are close to the corresponding IPCC default values. The broad distributions, with limited clustering, suggest substantial cross-country variability in the coal properties.

**Figure 1:**
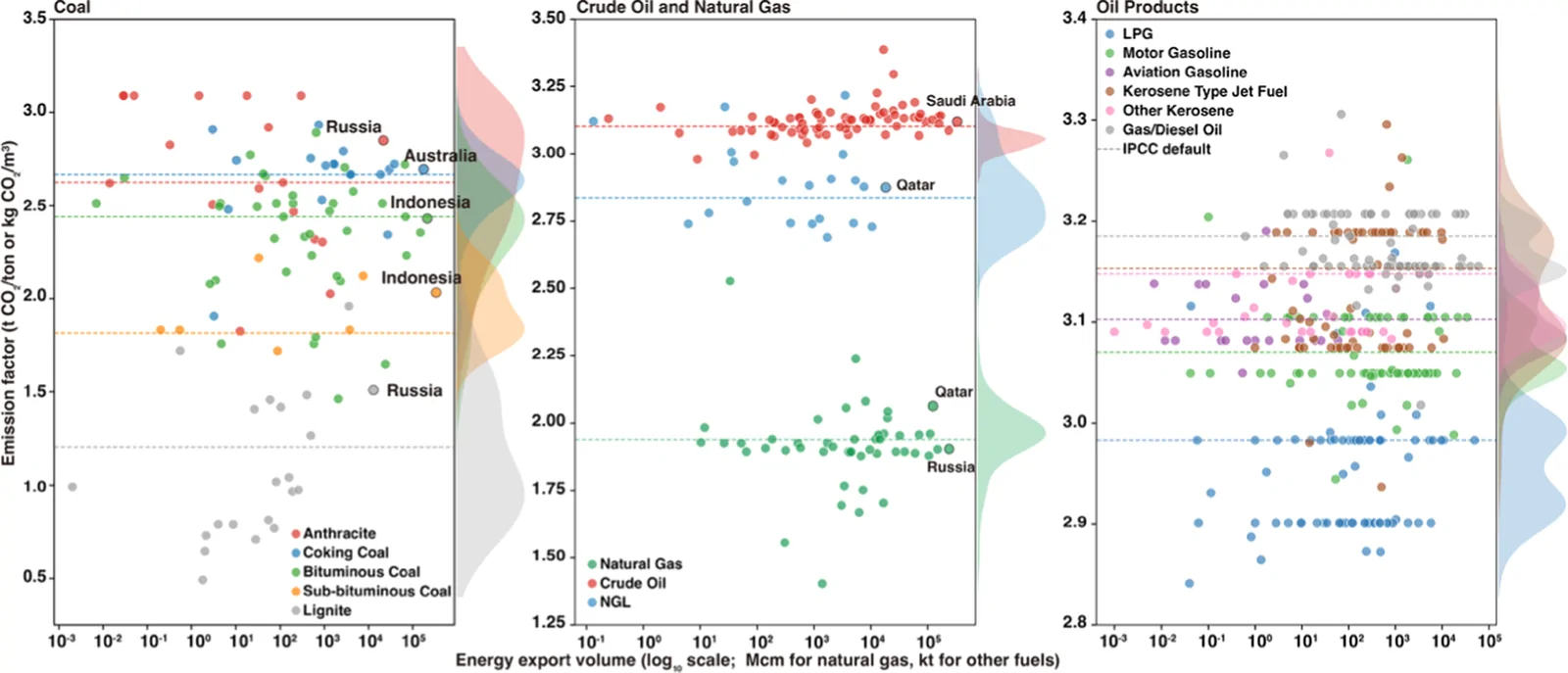
Emission factors (t CO_2_/t for solid and liquid fuels and kg CO_2_/m^3^ for natural gas) plotted against log_10_-scaled energy export volumes for selected fuels. Each dot represents a country’s emission factor for a specific fuel subtype, plotted against its export volume (log_10_ scale). Colors indicate fuel subtypes, and right-side kernel density plots show the distribution of emission factors. Emission factors are calculated based on country-specific net calorific values and available carbon content. Horizontal dashed lines indicate IPCC default values for each fuel type. For natural gas, the IPCC default reference value was first converted to a mass-based emission factor using the IPCC default net calorific value and carbon content. To match the volume-based natural gas emission factors, this value was further converted using an assumed natural gas density of 0.72 kg/m^3^. This density was used only to display the IPCC reference line.

Compared with coal, crude oil, NGL, and natural gas show much narrower emission-factor distributions. Although differences remain visible among major exporters, as illustrated by Qatar’s natural gas emission factor, countries with large export volumes, such as Russia and other Middle Eastern exporters, are generally located near the corresponding IPCC default reference lines.

Oil products exhibit the narrowest emission-factor distribution. This pattern is consistent with the fact that refining is designed to produce finished petroleum products that meet product-specific physical and chemical specifications, such as those for gasoline^14^ and diesel fuel.^13^ During refining, crude oil, which contains various hydrocarbons and impurities, is separated and further processed into distinct product fractions.^34^ As a result, country-specific differences in net calorific values and carbon contents are smaller for refined oil products, leading to a narrower range of emission factors.

### 3.2 Global Fossil Fuel Trade Pattern

In 2020, an estimated 17.5% of global coal production, 52.1% of global crude oil production, and 27.3% of global natural gas production were traded internationally, measured in physical units. On an energy-equivalent basis, these traded fossil fuels represented approximately 29.3% of the total primary energy supply, with crude oil having the largest trade volume among the primary fuels considered. Figure 2 shows net fossil fuel-related CO_2_ emission flows associated with trade among selected major importing and exporting countries and regions. In addition to primary fuels, namely, coal, crude oil, and natural gas, we also present the global trade flow of oil products because, based on our calculations of the 2020 data, 98.6% of the crude oil was ultimately consumed as refined petroleum products.

**Figure 2:**
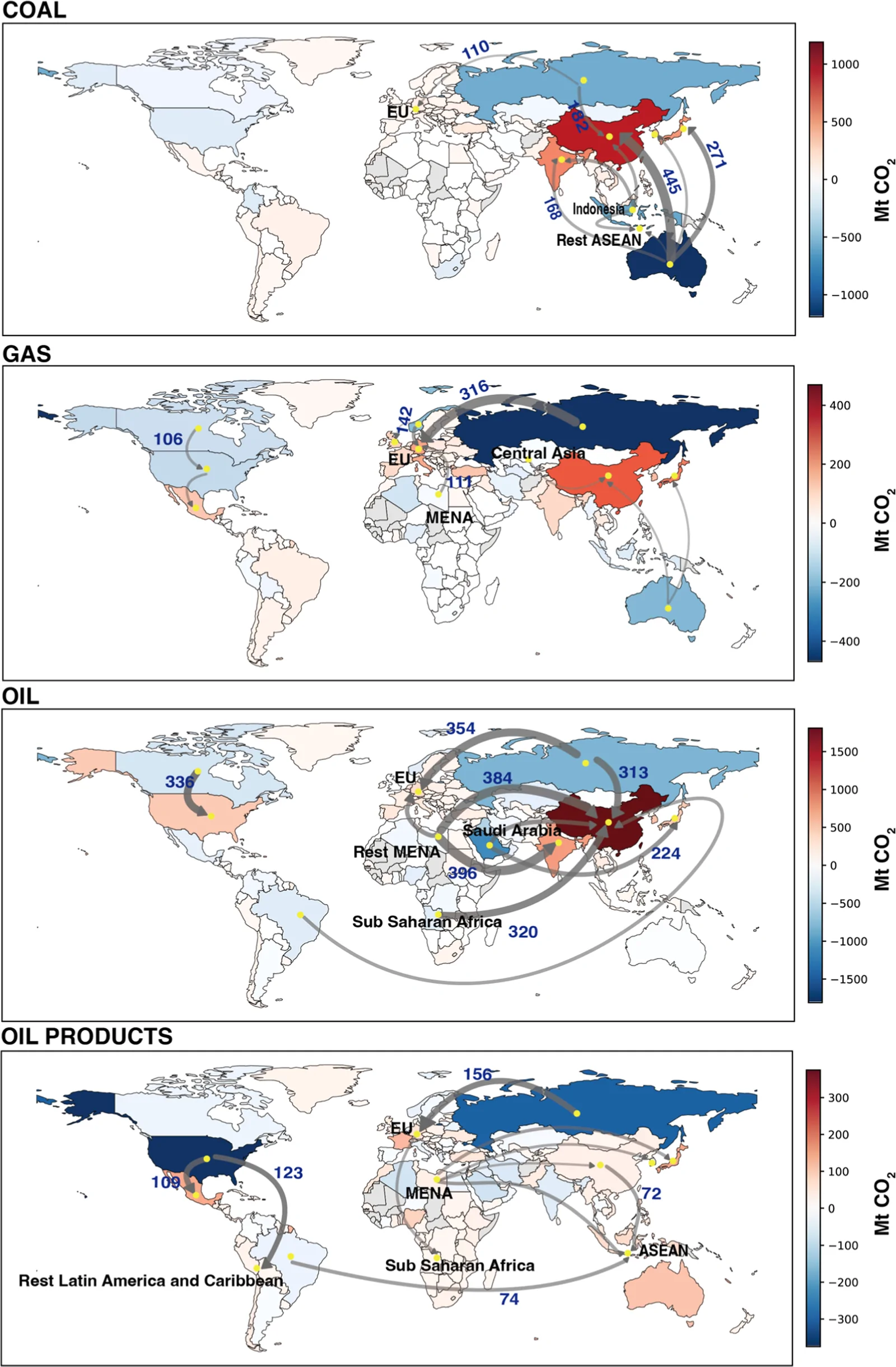
Net export flows of fossil fuel-related CO_2_ emissions in 2020. This figure presents the global net CO_2_ emission flows associated with the trade of coal, natural gas, crude oil, and refined oil products. Gross exports and imports are not shown separately. The color scale indicates the net direction of fossil fuel-related CO_2_ flows: red denotes net inflows of CO_2_ emissions, while blue denotes net outflows. Arrows show the major interregional emission transfers, and major flows are labeled in Mt CO_2_.

In general, fossil fuel trade is dominated by a few key players, with China, the EU, Japan, Korea, and India being major importers. Their emissions are influenced by the emission factors of fossil fuels from dominant exporters, such as the Middle East and North Africa (MENA) countries, Russia, Australia, and Indonesia. The United States, however, assumes a dual role as both a major importer and an exporter, reflecting its substantial domestic production and complex trade dynamics. Because Figure 2 presents net flows, countries with substantial two-way fossil fuel trade may not appear as major net exporters, even when their gross exports are large.

Each energy type exhibits distinct regional trade flows. Coal trade remains highly regionalized, with the Asia-Pacific region dominating both supply and demand. Australia (1192.0 Mt), Indonesia (630.4 Mt), and Russia (627.1 Mt) account for over 75% of traded coal-related CO_2_ emissions, with China (925.6 Mt) and India (490.3 Mt) importing nearly half of global traded coal-related CO_2_ emissions in 2020. Japan, South Korea, and Vietnam also play important roles. In contrast, natural gas trade exhibits a more integrated structure, influenced by both pipeline networks and liquefied natural gas (LNG) transport. Russia, the world’s largest net exporter of natural gas, accounted for the largest share of global net natural gas-related CO_2_ outflows in 2020, at 24.2% (417.3 Mt). Norway also remained a major net exporter, accounting for 9.6% (165.5 Mt), and together Russia and Norway served as key pipeline suppliers to meet European natural gas demand in 2020. Those shares have changed since the Russian–Ukrainian war.^35^ In Asia, major importers have diversified their LNG import portfolios, reducing reliance on individual suppliers.^36^ China illustrates the broader diversification of gas supply by combining pipeline imports from Central Asia, mainly Turkmenistan, with LNG shipments from Australia.

Crude oil trade is more globalized. While regional trade flows are dominant for some markets, such as Canada exporting most of its crude oil to the US and Russia supplying Europe, the overall crude oil trade network remains highly interconnected. The MENA region remains the core oil exporting region, accounting for 41.9% (2613.7 Mt) of global crude oil exports and supplying East Asia, South Asia, and Europe. In addition, Brazil is also a key supplier to Asia, particularly China. Beyond crude oil trade, the global market for oil products exhibits its own distinct dynamics, driven by refining capacity and trade hubs. The US, as the world’s largest refined oil exporter, accounts for 20.2% (or 433.8 Mt) of the global exports, with Mexico as its primary destination, alongside major markets in South America and North America. Similarly, the Netherlands and Singapore serve as redistribution hubs for refined oil products, supporting surrounding countries through their refining infrastructure and geographic advantage.

Global fossil fuel trade patterns are shaped by resource distribution, regional energy demand, energy infrastructure, and transport logistics. Coal resources are widely distributed.^37^ Global coal trade was traditionally split between the Pacific Basin, led by Japan and South Korea, and the Atlantic Basin, where Europe dominated imports.^38^ However, tightening environmental policies in Europe, including coal phase-out initiatives, have reduced European coal demand. Meanwhile, rising demand in China, India, and Southeast Asia has shifted coal trade toward the Asia-Pacific region.^38^ In contrast, crude oil and natural gas reserves are geographically concentrated, requiring long-distance exports to meet global demand. The expansion of the LNG infrastructure and pipeline networks has made natural gas trade more interconnected. Additionally, refining capacity and technical compatibility between crude oil types and refineries influence oil flows.^16,39,40^ Inland logistics, particularly for bulk coal, can significantly increase overall costs, sometimes accounting for up to 70% of the final price,^41^ and thereby create economic boundaries that shape trade routes. These factors collectively explain the differences in trade structures observed across coal, gas, oil, and oil products.

### 3.3 Reassessment of Global Emissions

Our study utilizes global fossil fuel trade data to trace each fossil fuel back to its country of extraction and applies origin-specific emission factors to reassess CO_2_ emissions from fossil fuel combustion across 150 countries and regions. In 2020, these emissions totaled approximately 34.0 Gt CO_2_, of which coal and coal products, oil and oil products, and natural gas accounted for 42.8%, 34.9%, and 22.2%, respectively. To evaluate the effect of origin-specific emission factors on global and national CO_2_ emission estimates, we constructed a nonorigin-traced control estimate using the same energy consumption data but combustion-country emission factors. This design isolates the effect of fuel-origin tracing by keeping energy consumption data unchanged and varying only the assignment of emission factors. The control estimate should not be interpreted as a strict IPCC Tier 1 benchmark based on IPCC default emission factors. Rather, it represents a nonorigin-specific combustion-country emission factor benchmark that does not distinguish between domestically produced and imported fuels. The nonorigin-traced control estimate was approximately 33.7 Gt CO_2_. Based on the unrounded estimates, it was 339.2 Mt (1%) lower than the origin-traced estimate. This difference is comparable to the fossil fuel combustion CO_2_ emissions of the United Kingdom in our 2020 estimate. The global discrepancy was predominantly driven by coal-related emissions, which accounted for 89.9% (304.8 Mt), while natural gas contributed 9.9% (33.5 Mt). Oil-related fuels made a limited contribution. This pattern indicates that the accounting deviation is determined not by trade volume alone, but by the combination of traded volume, origin-specific emission factor differences, and trade concentration. The sensitivity of this central estimate to country-specific emission factor variation was evaluated using the Monte Carlo analysis described in Section 3.4. This analysis yielded a range of 224.7 to 379.7 Mt CO_2_ (2.5th–97.5th percentile) for the global discrepancy. Although oil-related fuels are traded in large quantities, their contribution to the global discrepancy is limited because their emission factors vary less across exporting countries, especially for refined oil products manufactured to meet product-specific physical and chemical specifications. Coal shows the opposite pattern, with greater cross-country variation in emission factors and trade flows concentrated among a few major exporters, such as Indonesia and Russia. This combination explains coal’s 89.9% share of the global discrepancy. Figure 3 extends this comparison to selected countries by showing absolute changes by fuel type and relative changes in total combustion CO_2_ emissions.

**Figure 3:**
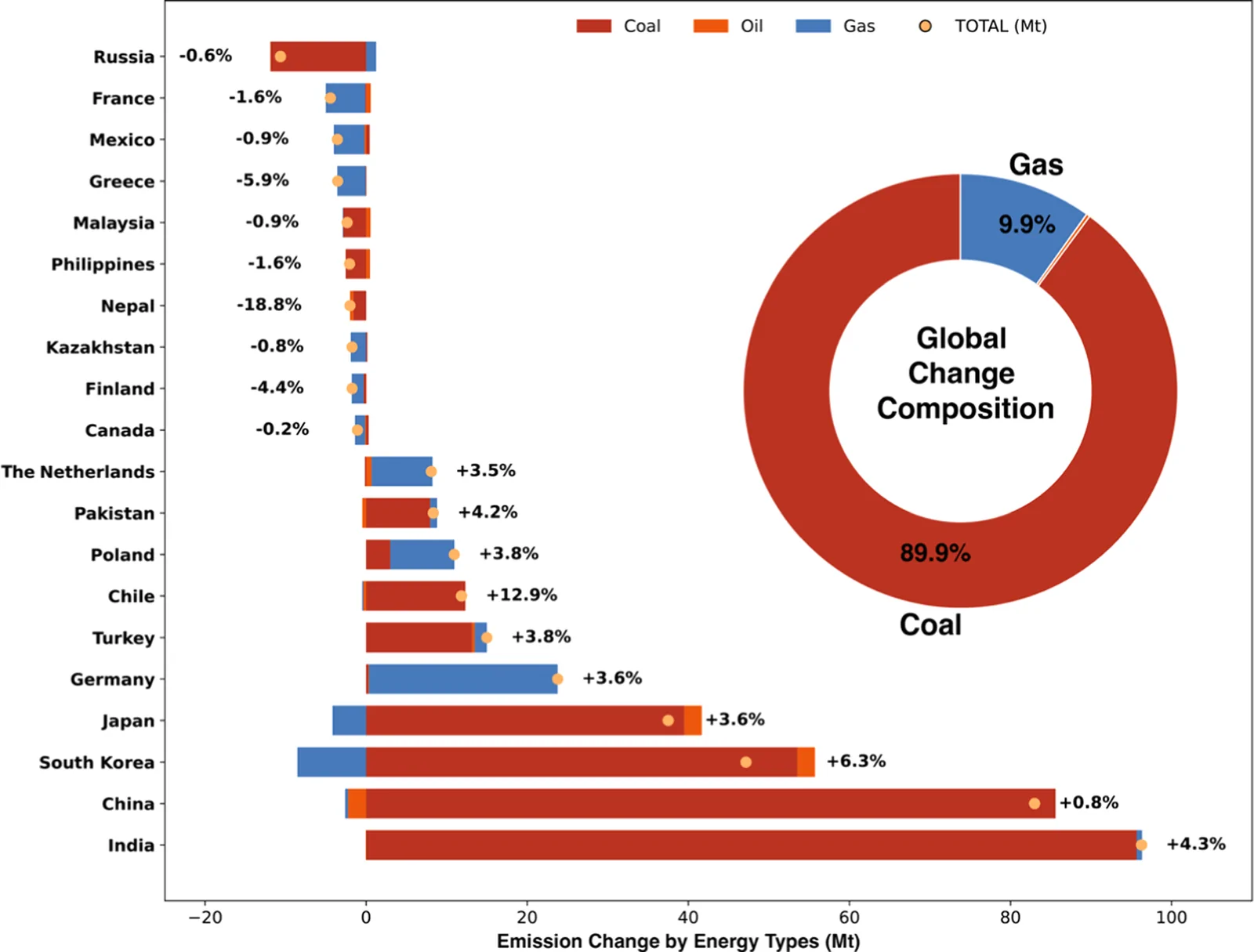
Country-level absolute and relative changes in combustion CO_2_ emissions between the origin-traced and nonorigin-traced control estimates for selected countries. Horizontal bars show changes by fuel type, calculated as the origin-traced estimate minus the control estimate. Yellow points indicate total changes, and the labels report relative changes in total emissions. The inset summarizes the fuel composition of the global discrepancy: coal and natural gas contribute 89.9% and 9.9%, respectively, whereas the oil-related contribution is too small to be visible.

Compared with the nonorigin-traced control estimate, 20 countries experienced an increase in emissions of more than 1%. Among countries with substantial absolute emission changes, Chile showed one of the largest relative increases, with total emissions of 91.9 Mt and a 37.4% rise in coal-related emissions. South Korea (743.9 Mt) and India (2249.0 Mt) showed similar patterns, where coal remained the dominant contributor, although South Korea’s increase was partially offset by a reduction in gas-related emissions. In Germany (651.3 Mt), Poland (285.3 Mt), and the Netherlands (233.2 Mt), the primary driver of emission growth was natural gas, with gas-related emissions increasing by 17.3%, 26.2%, and 10.2%, respectively.

A total of 19 countries show a decrease of more than 1%. France (275.6 Mt) experienced a 1.6% decrease, primarily due to lower natural gas-related emissions, while the Philippines (128.1 Mt) showed a similar relative decrease driven mainly by coal-related emissions. Other countries with notable relative decreases, such as Nepal, contributed less to the global discrepancy because of their relatively small emission baselines.

Emissions in 111 countries exhibit only minor changes (within ±1%). China (10851.4 Mt) increased by only 0.8%, but the absolute increment of 83.0 Mt was the second largest among all countries, surpassed only by India’s 96.3 Mt. This change was primarily driven by coal-related emissions but was partially offset by reductions in oil- and natural gas-related emissions. In countries with abundant fossil fuel resources, such as the United States (4650.1 Mt), Australia (396.0 Mt), and Russia (1794.7 Mt), changes were minimal.

At the regional level, we observe increased emissions across the Asia-Pacific region. This is particularly evident in countries such as India, South Korea, and Japan, which rely heavily on imported coal from Indonesia and Russia. These imports, associated with higher emission factors, result in higher CO_2_ emissions compared to the nonorigin-traced control estimate. In contrast, China consumes a mix of both imported and domestically produced coal, leading to a total increase in emissions. On the African continent, the majority of countries experienced a decrease in emissions, primarily driven by a reduction in oil-related emissions. In most African countries, the energy mix is dominated by refined oil products, which are largely imported from European countries with comparatively lower emission factors for refined oil products.

### 3.4 Robustness of the Emission Reassessment

We evaluated the robustness of the estimated global discrepancy under three sensitivity scenarios addressing emission factor uncertainty (scenario 1), bilateral trade allocation (scenario 2), and treatment of missing bilateral trade ratios (scenario 3).

In scenario 1, we conducted a targeted Monte Carlo analysis of emission factor uncertainty for selected major coal and natural gas exporters. Candidate country–fuel pairs were ranked from highest to lowest according to their 2020 physical export volumes. For each targeted fuel group, we first assembled a candidate set covering at least 80% of total physical export volume and then retained only pairs for which country-specific emission factor could be documented. The final literature-supported coal cases represent 83.7% of total export volume across the five primary types of coal, whereas the natural gas cases represent 73.4% of total natural gas export volume. Coal and natural gas together accounted for virtually all of the global discrepancy in the main analysis; crude oil and oil products contributed less than 1% and were excluded from scenario 1 because their emission factors exhibited lower cross-country variation. Variation is particularly limited for refined oil products, which are manufactured to meet product-specific physical and chemical specifications. In scenario 2, we tested sensitivity to the bilateral trade data used to assign the origins of imported fuels. In our main analysis, we used shares calculated from exporter-reported UN Comtrade export data to allocate each importing country’s fuel imports across source countries, whereas in this scenario, we used mirrored import shares calculated from importer-reported UN Comtrade import data instead. In scenario 3, we examined sensitivity to the treatment of missing bilateral trade ratios by replacing the substitute shares used in our main analysis with GTAP 2017 sectoral trade shares (scenario 3A) and fuel-specific global-average shares (scenario 3B). Detailed definitions, implementation procedures, and literature-supported emission-factor ranges and sources for scenario 1 are provided in Supporting Information, Section S1 and Table S1.

Across 10,000 Monte Carlo draws, the estimated global discrepancy ranged from 224.7 to 379.7 Mt CO_2_ (2.5th–97.5th percentile), with a median of 302.2 Mt CO_2_, compared with our estimate of 339.2 Mt CO_2_ (Figure 4). This interval reflects the sensitivity of the estimate to literature-supported emission-factor variation among the selected major exporters. The main estimate lies above the Monte Carlo median because the distributions for selected country–fuel pairs are centered on literature-based emission factors rather than on baseline factors. In particular, lower literature-based emission factors for Indonesian subbituminous and bituminous coal and Russian anthracite shift the estimated discrepancy downward. These effects outweigh the upward adjustments associated with South African bituminous coal and Australian and Norwegian natural gas. The lower and upper ends of the range therefore reflect alternative combinations of country-specific emission-factor adjustments among major exporters. Coal remained the dominant contributor in every draw, accounting for 77.2–89.8% of the discrepancy, while natural gas accounted for 9.9–22.5%.

**Figure 4:**
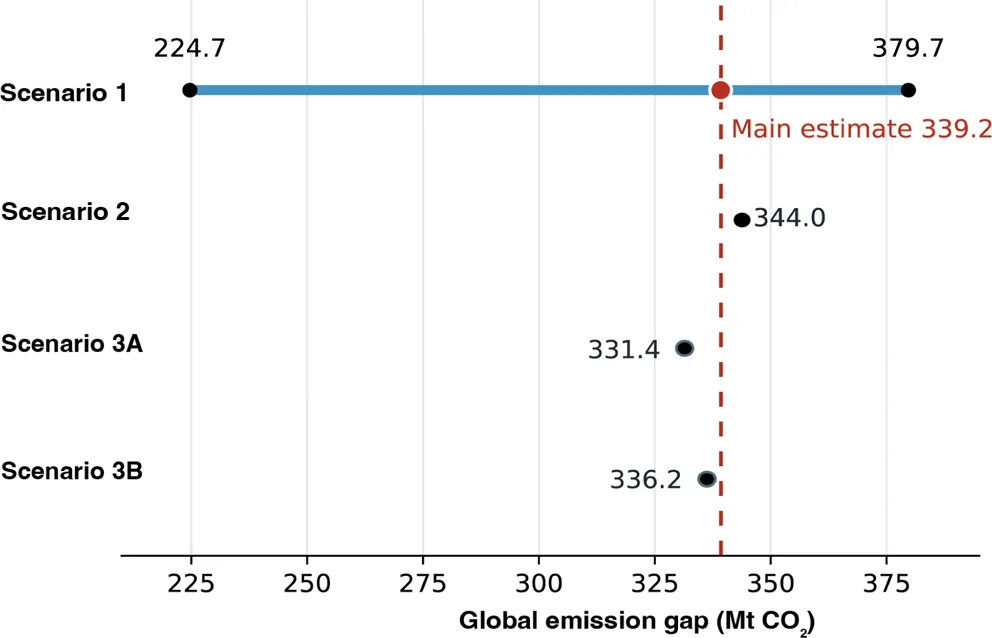
Robustness of the estimated global emission discrepancy under alternative uncertainty and sensitivity scenarios. The horizontal interval shows the 2.5th–97.5th percentile range of the Monte Carlo analysis for the emission factor in major coal- and natural-gas-exporting countries. The dashed line marks the estimate from the main analysis. Dots indicate the estimated discrepancy under the mirrored-import allocation scenario and under two alternative treatments of missing bilateral trade ratios using GTAP 2017 sectoral trade shares and fuel-specific global-average shares.

The trade allocation (scenario 2) and missing ratio (scenario 3) scenarios produced much smaller changes in the estimated discrepancy (Figure 4). Reallocating imported fuels using mirrored import shares increased the discrepancy by 4.8 Mt CO_2_, indicating that the main result was not strongly affected by whether bilateral fuel origins were assigned from exporter-reported or importer-reported trade data. Replacing missing bilateral trade ratios with GTAP 2017 sectoral trade shares or fuel-specific global average shares changed the discrepancy by −7.8 and −3.0 Mt CO_2_, respectively. These latter effects were limited because the replacements were concentrated mainly in oil-related fuels, which contributed little to the discrepancy and showed comparatively constrained cross-country variations in emission factors.

## 4 Discussion

Our results reveal a 339.2 Mt global discrepancy between the origin-traced estimate and the nonorigin-traced control estimate, a magnitude comparable to the United Kingdom’s fossil-fuel combustion CO_2_ emissions in 2020. This finding indicates that accounting for fuel origin can improve the accuracy of combustion CO_2_ estimates by assigning emission factors that better reflect the properties of the fuels actually consumed. This consideration becomes particularly important because the source-country composition of national fossil-fuel imports can change over time and may not be fully captured by emission factors that do not distinguish fuel origin.

This limitation is particularly relevant to IPCC Tier 1 methods, in which default emission factors are applied to national activity data.^7^ Consequently, the same emission factor is generally applied to a given fuel category within a country regardless of whether the fuel is domestically produced or imported. Although IPCC Tier 2 encourages the use of country-specific emission factors, preferably based on more disaggregated and measured fuel characteristics, such data are not always available for imported fossil fuels. National emission factors may therefore fail to reflect changes in the origin mix and fuel quality of the imported fuels. Generalized treatments without explicit fuel-origin tracing are also common in global emission data sets, including BP, CDIAC, EDGAR, and the Global Carbon Project. Such treatments are practical, especially for countries with limited data, but they can introduce discrepancies in national inventories, particularly in countries that rely heavily on fossil fuel imports.

In our analysis, this concern is most evident for coal, which accounts for 89.9% of the global discrepancy. Given that Australia, Indonesia, and Russia account for 76.2% of global coal exports, accurate emission factors from these major exporters are particularly important. These factors can affect the accuracy of the emission estimates for countries that rely heavily on imported coal, especially China, India, Japan, and South Korea in Asia as well as Germany and Poland in Europe.

Beyond these fuel-specific drivers, the distinction between relative and absolute discrepancies is also important for interpreting national-level results. Relative changes indicate the sensitivity of a country’s emissions to the choice of emission factors, while absolute changes show the magnitude of its contribution to the global discrepancy. China provides a clear example. Although its relative increase is only 0.8% due to its large emissions baseline, the corresponding absolute increase reaches 83.0 Mt CO_2_, making it one of the largest national adjustments in our results. Therefore, both relative and absolute discrepancies should be considered when assessing the implications of origin-specific emission factors for national inventories and global emission accounting.

These discrepancies have broader implications for international climate policy. Several countries with substantial emission discrepancies have also adopted ambitious mitigation targets. For instance, India aims to reduce emission intensity by 45% from 2005 levels by 2030, while Germany and Poland are covered by the EU-wide goal of reducing net greenhouse gas emissions by at least 55% from 1990 levels by 2030.^42,43^ Whereas carbon leakage through embodied emissions in traded goods has been widely discussed,^44^ comparatively less attention has been paid to origin-related emission factor mismatches within complex fossil-fuel supply chains. Such mismatches can occur when imported fuels are assigned emission factors that do not reflect their extraction origin. The fuel origin remains relevant even within territorial inventory accounting because it can affect the appropriate emission factor assigned at the point of combustion. If emission baselines are misrepresented because of inappropriate emission factors, assessments of mitigation progress may also be affected. Greater alignment between inventory methods and actual fuel trade structures could therefore improve the accuracy and credibility of NDC tracking and support the global stocktaking under the Paris Agreement.

These policy implications should be interpreted within the system boundary of our analysis, which covers only CO_2_ emissions released during fossil fuel combustion. Tracing fossil fuels to their countries of extraction is used to improve the assignment of combustion emission factors rather than to estimate emissions from extraction, processing, transportation, or distribution. Upstream fugitive methane emissions from coal mining and oil and gas systems, including episodic superemitting events, are outside the system boundary, as are CO_2_ emissions from nonenergy uses of fossil fuels. The exclusion of upstream methane is particularly relevant when interpreting the coal- and natural gas-related results because they reflect combustion CO_2_ accounting only.

Because of data limitations, our estimates also rely on several assumptions, including the use of IPCC default values for carbon content and oxidation rates where country-specific measurements are unavailable and the assumption of homogeneous mixing between domestic and imported fuels in transformation processes. Residual uncertainty may also remain in source attribution when fuels transit through intermediary countries, but the corresponding trade flows are not explicitly reported as re-exports. The results should therefore be interpreted in light of these assumptions and the data limitations.

The results should also be interpreted as a 2020 snapshot rather than as a long-term average of fossil-fuel trade patterns. We selected 2020 because, at the time of data compilation, it provided the most complete and consistently harmonized data available across the data sets used in this analysis. Fossil fuel trade patterns are dynamic, and 2020 was also affected by unusual disruptions in energy demand and international trade. Therefore, country-level origin shares and the resulting emission discrepancies may vary across the years. Nevertheless, the approach developed here is not specific to 2020 and can be extended to other years as consistent trade, energy, and emission factor data become available.

Beyond the system boundary, modeling, and temporal limitations described above, the availability of emission factor data remains a challenge. Emission factors are derived from net calorific value, carbon content, and oxidation factor, but publicly accessible country-specific information for these parameters is uneven across fuels and countries. Although the IPCC Emission Factor Database (EFDB) is one of the most comprehensive freely accessible sources, some country-specific records are still based on relatively old reference materials. For example, the Australia-specific coking coal net calorific value identified in the IPCC EFDB is recorded as a 1990 country-specific value with a technical reference to OECD/IEA (1993),^26^ whereas more recent official Australian factors are available from national sources.^45^ Similarly, carbon content data are available for only a limited number of countries, and oxidation factors are often assumed to be 1 because of insufficient data. The IEA provides more frequently updated net calorific value data than the IPCC EFDB, but its updates rely on submissions from national administrations and do not follow a consistent schedule across the countries. Even where country-specific net calorific values are updated every 5–10 years, such updates may still be insufficient to fully capture changes in fuel characteristics over time.

Regularly updating country-specific emission factors is technically demanding and resource intensive, which can hinder timely revisions. For example, determining representative coal net calorific values requires both measurements by specialized laboratories and national-level data aggregation across mines and producers.^46^ The scale of this task is illustrated by Liu and co-workers (2015), who combined measurements from 602 coal samples across 100 major coal-mining areas with production statistics from 4243 coal mines when developing updated emission factors for coal in China.^9^

One potential approach to address this challenge is to integrate fuel quality parameters, such as net calorific value and carbon content, into the trade documentation of fossil fuels. Fuel producers, such as mining companies and refineries, routinely measure calorific values and other quality attributes for product specification and pricing.^46^ Renewable energy certificate (REC) systems provide a useful precedent for such approaches. In these systems, each REC records information on the generation source, facility location, and emission factor for 1 MWh of renewable electricity, allowing these attributes to be tracked through a certificate registry.^47^ A similar structure could link reported fossil-fuel quantities to their extraction origins and corresponding net calorific value and carbon content. Importing countries could then aggregate reported fuel quantities by origin and calculate quantity-weighted averages of the corresponding quality parameters for use in national inventories. This approach could make emission factor assignment more representative while using existing reporting channels and limiting additional data-collection burdens.

Nevertheless, wider implementation of this approach to reporting fuel origin and fuel quality information would require collaboration among fossil-fuel importers, exporters, and producers. Detailed supplier-specific information may also be commercially or strategically sensitive. An intermediate option would be to establish benchmark fuel-quality profiles for emissions accounting. Related benchmark systems are already used for pricing in fossil fuel markets. For example, commercial coal benchmarks define reference products using standardized specifications, including net calorific value, sulfur content, ash content, and moisture.^48^ For emissions accounting, such a framework could define a limited set of benchmark fuel quality profiles for each fossil-fuel type, each characterized by representative net calorific value and carbon content. Using available quality information, importing countries could assign reported fuel quantities to the closest profile and combine the profile’s representative values with the applicable oxidation factor to calculate the corresponding emission factor. Because fossil fuels assigned to the same profile may still differ in the parameters underlying their emission factors, applying one representative factor to each profile would not capture all within-profile variation. Even so, distinguishing multiple quality profiles could yield more accurate emission estimates than generalized default-factor treatments that apply a common factor within each fuel category. This intermediate approach could also make data sharing more feasible by reducing the need to disclose commercially or strategically sensitive supplier-specific information.

Overall, our results show that the extraction origin of traded fossil fuels can affect combustion CO_2_ accounting. The global discrepancy is modest in percentage terms but substantial in absolute terms, and it is highly uneven across fuels and countries. This pattern reflects the combined effects of trade volume, emission factor variability, and trade concentration, with coal being the dominant driver because it combines all three factors. These findings highlight the value of assigning emission factors that better reflect fuel properties associated with the extraction origin. More regular reporting and updating of fuel-quality data by the country of origin could improve national inventories and strengthen mitigation tracking under the Paris Agreement.

## Supplementary Material







## Notes

Origin-traced 2020 combustion CO_2_ emission matrices by fuel group, origin country, and combustion country, with fuel-category mappings (XLSX)
IEA-HS mappings, imputation flags, and country-level fuel-origin shares used for trade-origin assignment (ZIP)
Design and results of the sensitivity analyses, literature-supported emission-factor inputs, accounting-workflow schematic, and illustrative crude-oil and refined-product accounting example (PDF)
