# Supplementary material for: Global Combustion CO2 Accounting Neglects the Origin of Traded Fossil Fuels
Source: Environ Sci Technol. 2026 Aug 27;60(36):25385–96. doi: 10.1021/acs.est.6c01630 (PMC13585052; doi:10.1021/acs.est.6c01630)
Supplement: Supplementary file 1 [file es6c01630_si_003.pdf]

Supplementary Materials for

Global combustion CO_2 accounting neglects the origin of traded fossil fuels

Binyuan Liu[1], Yuli Shan[2*], Yuru Guan[2], Steven J. Davis[3], Ye Hang[2], Liang Jing[4], Xiande Ji[1], Wenqiang Wang[5], Klaus Hubacek[1*]

1. Energy and Sustainability Research Institute Groningen, University of Groningen, Groningen 9747 AG, the Netherlands

2. School of Geography, Earth and Environmental Sciences (GEES), University of Birmingham, Birmingham B15 2TT, UK

3. Department of Earth System Science, Stanford University, Stanford, CA 94305, USA

4. Energy Sustainability Analysis, Technology Strategy and Planning Department, Saudi Aramco, Dhahran 31311, Saudi Arabia

5. Institute of Blue and Green Development, Shandong University, Weihai 264209, China

*Corresponding author. Email: y.shan@bham.ac.uk (Yuli Shan); k.hubacek@rug.nl (Klaus Hubacek)

This PDF file includes:

Text S1. Robustness of the emission reassessment
Text S2. Illustration of the hybrid accounting framework
Table S1. Literature-supported inputs used in Scenario 1
Table S2. Example of combustion CO_2 accounting for crude oil and refined oil products.
Figure S1 Schematic overview of the accounting workflow

Other Supplementary Materials for this manuscript include the following:

Data S1. Origin-traced combustion CO_2 emission matrices by fuel group and country, 2020. (separate file)

This Excel file contains fossil-fuel-related combustion CO_2 emission matrices for 150 countries and regions. Each worksheet corresponds to a fossil-fuel product or aggregated fuel group used in the reported results. Rows represent the countries or regions of fuel origin, and columns represent the countries or regions where the fuels are combusted. Values are reported in Mt CO_2 for 2020. The `categories` worksheet documents how detailed IEA fuel products are aggregated into the reported fuel groups.

Data S2. Trade-origin assignment tables. (separate file)

This archive contains three machine-readable CSV files documenting the trade-origin assignment workflow. `IEA_HS_mapping.csv` reports the mapping between IEA fossil-fuel product categories and HS codes used to extract UN Comtrade data. `imputation_flags.csv` identifies country-fuel cases for which direct 2020 bilateral trade shares were unavailable and records the replacement rule used. `country_level_origin_shares.csv` reports the final origin shares by fuel, origin country, and destination country used in the main analysis.

S1. Robustness of the emission reassessment

This section provides methodological details and extended results for the sensitivity analyses summarized in the main text.

Scenario 1: Emission-factor uncertainty

1.1 Rationale and scope

Emission factors are determined by net calorific value, carbon content, and oxidation factor, each of which may vary across fuel origins. We therefore conducted a targeted sensitivity analysis to assess whether variation in these components would materially affect the estimated discrepancy. The analysis focused on coal and natural gas because these fuels accounted for nearly all of the discrepancy in the main results, whereas crude oil and oil-related fuels contributed little and have comparatively less variable emission factors than coal.

Given the limited availability of country-specific literature, we focused on major fuel-exporting countries for which such information could be found. Overall, despite the limited availability of country-specific literature ranges, the data used in the analysis covered 83.7% of the coal-export volume and 73.4% of the natural-gas-export volume in the analysis.

1.2 Literature inputs and construction of uncertainty ranges

We used four approaches to construct the country-specific emission factor ranges reported in Table S1. First, for cases constructed from separate net calorific value and carbon-content inputs, the lower bound, mode, and upper bound of the emission factor were calculated by combining the corresponding NCV and carbon-content values. Country-specific values were used where available, while missing bounds were supplemented with fuel-specific IPCC ranges or explicitly identified proxy values. For example, the published values for Indonesian bituminous coal yielded emission factors of 2.251, 2.280, and 2.317 t CO_2 per t fuel, respectively [4]. For Russian bituminous coal (case 3), the net calorific value reported for coal from the Kuznetsk Basin (Kuzbass), a major Russian coal-producing region, was used as the mode [2], while the IPCC bituminous coal net calorific value bounds were used as the lower and upper values [1]. These values were combined with carbon content values derived from the Russian national inventory [2], yielding lower, modal, and upper emission factors of 1.877, 2.397, and 2.882 t CO_2 per t fuel, respectively. This approach was applied to cases 1, 3, 4, and 8–10 in Table S1.

Second, for LNG cases where a range of gross calorific values was reported but no compatible country-specific carbon content range was available, the gross calorific values were converted to approximate net calorific values using a factor of 0.9 and combined with the IPCC default natural-gas carbon content [1]. For Qatari LNG, applying this procedure to the reported gross calorific value range [7] produced lower, modal, and upper emission factors of 1.991, 2.005, and 2.094 kt CO_2/Mcm, respectively. This GCV-based approach was applied to cases 13, 17, and 18 in Table S1.

Third, where only a single country-specific net calorific value was available but compatible country-specific carbon content information was not, it was combined with the IPCC default

carbon content to calculate the modal emission factor. The IPCC lower-to-default and upper-to-default emission-factor ratios were then applied to this mode to construct the corresponding bounds. For Norwegian natural gas, the official export net calorific value of 36.60 TJ/Mcm [8] produced a modal emission factor of 2.053 kt CO_2/Mcm. Applying the IPCC natural-gas ratios produced lower and upper bounds of 1.987 and 2.134 kt CO_2/Mcm, respectively [1]. This approach was applied to case 14 in Table S1.

Fourth, where a directly usable country-specific emission factor was reported, that value was used as the mode. Source-specific lower and upper values were used where available. Otherwise, the relative width of the corresponding IPCC default emission factor range was applied to the country-specific mode. For other bituminous coal, for example, the IPCC reports lower, default, and upper values of 89,500, 94,600, and 99,700 kg CO_2/TJ, respectively [1]. We retained the lower-to-default and upper-to-default ratios of this range and applied them to the Australian-specific factor of 2.431 t CO_2 per t fuel [3]. This produced lower and upper bounds of 2.300 and 2.562 t CO_2 per t fuel, respectively. This approach was applied to cases 2, 5–7, 11, 12, 15, and 16 in Table S1.

Because country-specific oxidation factor ranges were not consistently available, we used general IPCC-based ranges of 0.98-1.00 for coal and 0.995-1.00 for natural gas. In the Monte Carlo analysis, these lower, central, and upper values were represented by triangular distributions. We performed 10,000 Monte Carlo draws, perturbing the 18 literature-supported country-fuel cases simultaneously while holding all other country-fuel cases and physical energy flow matrices at their main-analysis values. The detailed data sources and range-construction methods for each country-fuel case are summarized in Table S1.

Table S1. Literature-supported inputs used in Scenario 1.

Case	Fuel	Country	Input basis	Lower	Mode	Upper	Range construction	Main source(s)
1	ANTCOAL	Russia	NCV + CC	2.038	2.273	2.734	Russian official values with IPCC lower limit	Russian NIR [2]; IPCC [1]
2	BITCOAL	Australia	Direct EF	2.300	2.431	2.562	Australian official EF with IPCC proportional uncertainty range	Australia NGA Factors [3]; IPCC[1]
3	BITCOAL	Russia	NCV + CC	1.877	2.397	2.882	Russian official values with IPCC bituminous-coal NCV bounds	Russian NIR [2]; IPCC [1]
4	BITCOAL	Indonesia	NCV + CC	2.251	2.280	2.317	Published NCV and CC ranges	Damayanti and Khaerunissa [4]
5	BITCOAL	South Africa	Direct EF	2.057	2.590	3.123	Measured EF range from published sample variability	Kornelius et al. [5]
6	COKCOAL	Australia	Direct EF	2.545	2.757	2.944	Australian official EF with IPCC proportional uncertainty range	Australia NGA Factors [3]; IPCC[1]
7	COKCOAL	USA	Direct EF	2.511	2.721	2.905	US official EF with IPCC proportional uncertainty range	EPA EF Hub [6]; IPCC[1]
8	COKCOAL	Russia	NCV + CC	2.264	2.397	2.929	Russian official values with IPCC NCV bounds	Russian NIR [2]; IPCC [1]
9	LIGNITE	Russia	NCV + CC	1.299	1.568	2.214	Russian official values with IPCC upper limit	Russian NIR [2]; IPCC [1]
10	SUBCOAL	Indonesia	NCV + CC	1.842	1.879	1.906	Published NCV and CC ranges	Damayanti and Khaerunissa [4]
11	NATGAS	Russia	NCV + country/IPCC EF range	1.836	1.840	1.972	Russian country-specific EF as the mode, with IPCC EF bounds converted using the same Russian NCV	Russian NIR [2]; IPCC [1]
12	NATGAS	USA	Direct EF	1.861	1.923	1.998	US official EF with IPCC proportional uncertainty range	EPA EF Hub [6]; IPCC[1]
13	NATGAS	Qatar	GCV + IPCC default CC	1.991	2.005	2.094	Qatar LNG GCV range converted to an approximate NCV range; IPCC default natural gas CC	MARCOGAZ LNG Database [7]; IPCC [1]
14	NATGAS	Norway	NCV + IPCC EF range	1.987	2.053	2.134	Norwegian export NCV combined with the IPCC natural gas EF range	Norway NID[8]; IPCC[1]
15	NATGAS	Australia	Direct EF	1.955	2.020	2.099	Australian official EF with IPCC proportional uncertainty range	Australia NGA Factors [3]; IPCC[1]
16	NATGAS	Canada	Direct EF	1.915	1.962	1.966	Canadian official EF range	Canada ECCC [9]
17	NATGAS	Algeria	GCV + IPCC default CC	2.009	2.056	2.107	Algerian LNG GCV range converted to an approximate NCV range; IPCC default natural gas CC	MARCOGAZ LNG Database [7]; IPCC [1]
18	NATGAS	Nigeria	GCV + IPCC default CC	2.074	2.091	2.116	Nigerian LNG GCV range converted to an approximate NCV range; IPCC default natural gas CC	MARCOGAZ LNG Database [7]; IPCC [1]

Notes: Coal emission factors are expressed in t CO_2/t fuel, and natural-gas emission factors in kt CO_2/Mcm. For LNG cases based on reported gross calorific values (GCVs), net calorific values (NCVs) were approximated as 0.9 × GCV, and emission factors were calculated using the IPCC default natural-gas carbon content of 15.3 kg C GJ^{-1} and the molecular-weight ratio of 44/12. "IPCC proportional range" indicates that the country-specific central value was retained and the lower-to-default and upper-to-default ratios of the corresponding IPCC range were applied to it. "IPCC lower/upper bound" indicates that the corresponding IPCC endpoint was used when a country-specific bound was unavailable.

1.3 Results

Across 10,000 Monte Carlo draws, the estimated global discrepancy ranged from 224.7 to 379.7 Mt CO_2 (2.5th-97.5th percentile), with a median of 302.2 Mt CO_2. The point estimate from the main analysis, 339.2 Mt CO_2, lies within this interval. Coal remained the dominant contributor in every draw, accounting for 77.2-89.8% of the discrepancy, while natural gas accounted for 9.9-22.5%.

The lower median mainly reflects downward shifts in several high-leverage cases, especially Indonesian sub-bituminous and bituminous coal, and Russian anthracite. These downward shifts outweighed the upward shifts for South African bituminous coal and Australian and Norwegian natural gas. Although the literature-based ranges broadened the possible magnitude of the discrepancy, they did not alter its interpretation. Across the full uncertainty range, the estimated discrepancy remained substantial and was consistently dominated by coal.

Scenario 2: Mirrored-import trade allocation

In the main analysis, bilateral imports were constructed from exporter-reported trade shares, as described in Eq. S1 of the Methods:

$$\boldsymbol{Imports^{r\to j,i}} = \boldsymbol{IEA_{imp}^{j,i}} \times (\boldsymbol{Exports^{r\to j,i}} / \sum \boldsymbol{Exports^{j,i}}) \qquad \textbf{Eq. S1}$$

where $Imports^{r\to j,i}$ indicates the imports of energy type i from country r to country j. $IEA_{imp}^{j,i}$ refers to country j's total imports of energy type i, based on the data from IEA. $Exports^{r\to j,i}$ represents the export of energy type i from country r to country j. $\sum Exports^{j,i}$ represents that all the exports of energy type i to country j.

This approach allocates the total imports of energy type i in country j using the export shares reported by source countries. To test sensitivity to the choice of reporter side, we constructed an alternative import matrix using mirrored-import data reported by importing countries. In this scenario, the export-share term in Eq. S1 was replaced by the corresponding mirrored-import share:

$$\boldsymbol{Imports^{r\to j,i}} = \boldsymbol{IEA_{imp}^{j,i}} \times (\boldsymbol{UN\_imp^{r\to j,i}} / \sum \boldsymbol{UN\_imp^{j,i}}) \qquad \textbf{Eq. S2}$$

where $UN\_imp^{r\to j,i}$ denotes the import of energy type i reported by country j from country r after removing re-imports, and $\sum UN\_imp^{j,i}$ denotes the total import of energy type i reported by country j in the UN Comtrade database. Thus, country-level import totals from IEA were held constant, and only the allocation of those imports across source countries was changed. Consistent with the main analysis, natural gas was not included in this sensitivity test because its bilateral trade allocation was based on a separate IEA bilateral natural-gas trade dataset rather than the generic UN Comtrade workflow.

Replacing exporter-reported shares with mirrored-import shares changed the estimated global discrepancy by +4.8 Mt CO2 relative to the main result, equivalent to 1.42% of the estimated gap and 0.014% of total global emissions. This small change indicates that the alternative allocation did not materially alter the original structure of the major fuel flows driving the discrepancy, and that the main result is therefore not strongly sensitive to whether bilateral fuel origins are allocated from exporter-reported or importer-reported trade data.

Scenario 3: Replacement rules for missing trade ratios

Most bilateral origin shares used in the main analysis were derived directly from observed UN Comtrade export data. However, for a subset of exporter-fuel cases, the 2020 bilateral trade ratio was missing and was therefore replaced using documented substitute rules in the main analysis. These replacement cases represented 0.1% of total global trade in coal and coal products, 18.1% for oil and oil products, and 1.4% for gaseous fuels. The detailed case information is listed in Data S2. To test whether these substitutions materially affected the results, we constructed two alternative scenarios in which only these replaced shares were changed. These alternatives were not intended to represent preferred reconstructions of missing trade ratios, but to test whether the main result was sensitive to materially different replacement rules.

In Scenario 3A, the replaced shares were reassigned using GTAP 2017 bilateral trade shares wherever possible. Under this alternative, the global discrepancy decreased by 7.8 Mt CO_2, equivalent to 2.31% of the estimated gap and 0.023% of total global emissions. In Scenario 3B, they were reassigned using fuel-specific global-average export shares calculated from UN Comtrade data for the same fuel category. This alternative reduced the global gap by 3.0 Mt CO_2, equivalent to 0.90% of the estimated gap and 0.009% of total global emissions. Both alternatives, therefore, had only a minor effect on the estimated global discrepancy.

S2. Illustration of the hybrid accounting framework

2.1. Schematic overview of the accounting workflow

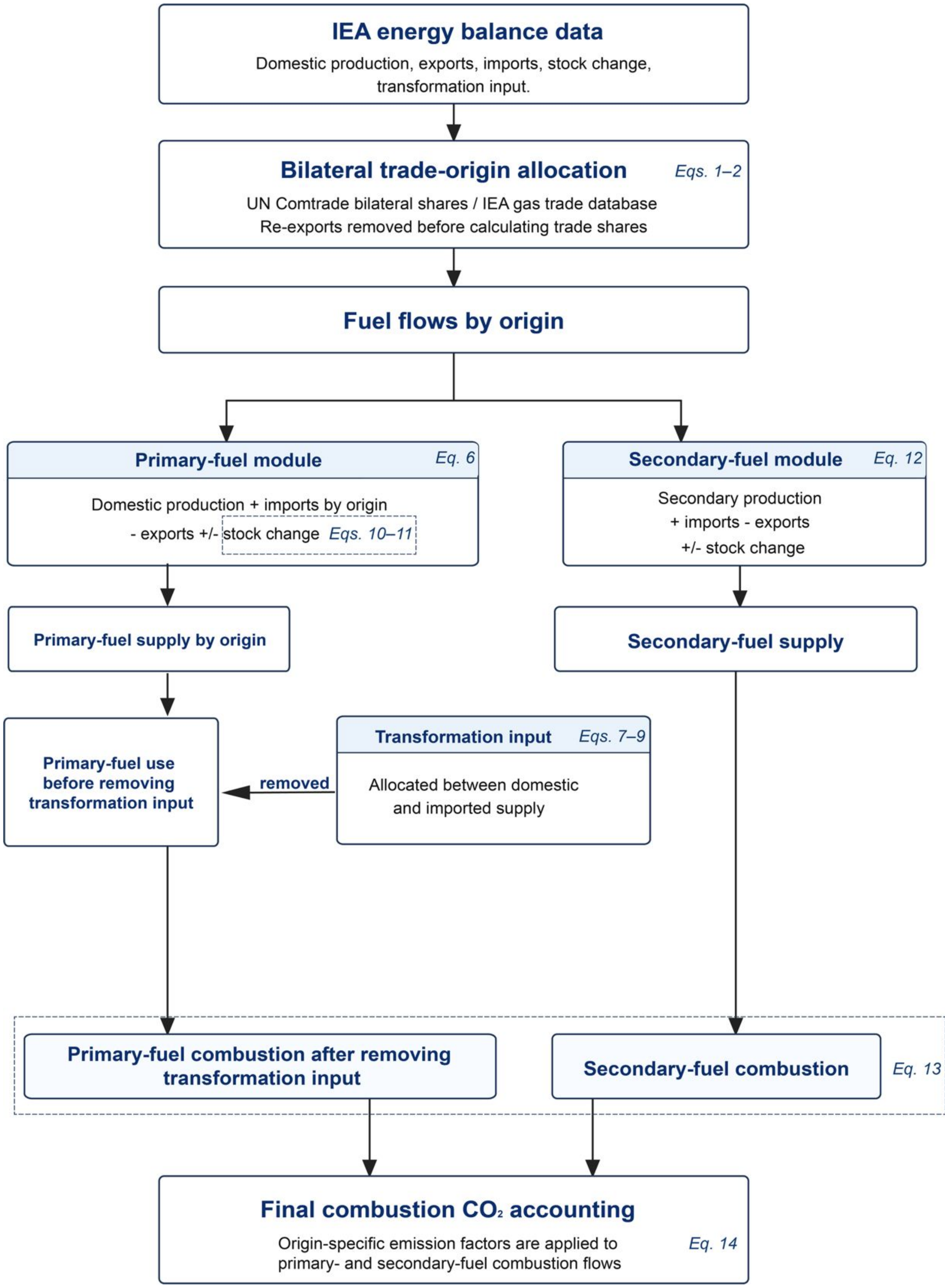

Figure S1 Schematic overview of the accounting workflow. Note: The primary- and secondary-fuel combustion flows are combined according to Eq. 5, with the expanded balance given in Eq. 13. Eqs. 10–11 apply to stock increases; fuels withdrawn from stocks are assumed to have been domestically produced. The same treatment is applied to secondary fuels.

2.2 Example of crude oil and refined oil products

Consider a country that produces 20 kt of crude oil domestically, imports 80 kt of crude oil, exports 10 kt, builds 5 kt of crude oil stocks, and uses 60 kt of crude oil as refinery input. Of the imported crude oil, 50 kt originates from country A and 30 kt from country B. This country also separately reports 55 kt of refined oil product production, of which 20 kt is exported. Imports and stock changes of refined oil products are assumed to be zero in this illustrative example. The detailed calculation processes are shown in **Table S2.**

Table S2. Example of combustion CO_2 accounting for crude oil and refined oil products.

Step	Calculation	Result	Eq(s).
1. Crude oil supply	Domestic supply after exports: 20 − 10 = 10 kt<br>Imported supply: 80 kt<br>Total supply: 10 + 80 = 90 kt	10 kt domestic;<br>80 kt imported	
2. Apparent crude-oil consumption after stock build-up	20 + 80 − 10 − 5	85 kt	6
3. Allocate the 5 kt stock build-up	Imported share of crude-oil supply: 80/90<br>Domestic: 5 × (1-80/90) = 0.56 kt<br>Imported: 5 × (80/90) = 4.44 kt<br>Country A: 4.44 × (50/80) = 2.78 kt<br>Country B: 4.44 × (30/80) = 1.67 kt	0.56 kt domestic;<br>2.78 kt from A;<br>1.67 kt from B	7,<br>10–11
4. Allocate the 60 kt refinery input	Domestic: 60 × (1-80/90) = 6.67 kt<br>Imported: 60 × (80/90) = 53.33 kt<br>Country A: 53.33 × (50/80) = 33.33 kt<br>Country B: 53.33 × (30/80) = 20.00 kt	6.67 kt domestic;<br>33.33 kt from A;<br>20.00 kt from B	7–9
5. Crude oil after stock build-up and refinery input remaining in the primary-fuel combustion	85-60	25 kt	
6. Crude oil after stock build-up and refinery input remaining in the primary-fuel combustion by countries	Domestic: 10 − 0.56 − 6.67 = 2.78 kt<br>Country A: 50 − 2.78 − 33.33 = 13.89 kt<br>Country B: 30 − 1.67 − 20.00 = 8.33 kt	2.78 kt domestic;<br>13.89 kt from A;<br>8.33 kt from B	13
7. Refined oil product consumption in the refining country	Secondary production + imports − exports ± stock change<br>= 55 + 0 − 20 + 0	35 kt	12
8. Combine the primary- and secondary-fuel terms	Retain 25 kt of crude oil and 35 kt of refined oil products as separate fuel-specific combustion flows.	25 kt crude oil;<br>35 kt refined products	5, 13

Step	Calculation	Result	Eq(s).
9. Calculate combustion CO_2 emissions	Multiply each origin-specific crude-oil flow and the refined-oil-product flow by its corresponding fuel- and origin-specific emission factor.	Total combustion CO_2 emissions	14

In this example, 5 kt of the 90 kt crude oil supply is added to stocks and 60 kt is used as refinery input, leaving 25 kt in the primary-fuel combustion term. The 60 kt refinery input is not counted as crude-oil combustion. Instead, the separately reported 55 kt of refined oil products enters the secondary-fuel module, with 35 kt counted in the refining country and 20 kt counted in the importing countries. This treatment ensures that the crude oil refinery input and the resulting refined products are not both counted as combustion emissions.

Reference:

(1) Garg, A.; Kazunari, K.; Pulles, T. *2006 IPCC Guidelines for National Greenhouse Gas Inventories, Volume 2: Energy, Chapter 1*; 2006; https://www.ipcc-nggip.iges.or.jp/public/2006gl/vol2.html (accessed 2025-02-24).
(2) Russian Federation. *2025 National Inventory Document (NID)*; 2025. https://unfccc.int/documents/646536 (accessed 2026-05-18).
(3) DCCEEW. *National Greenhouse Accounts Factors: 2021 - DCCEEW*; 2021. https://www.dcceew.gov.au/climate-change/publications/national-greenhouse-accounts-factors-2021 (accessed 2026-05-18).
(4) Damayanti, R.; Khaerunissa, H. Carbon Dioxide Emission Factor Estimation from Indonesian Coal. *IMJ* **2018**, *21* (1), 45–58. https://doi.org/10.30556/imj.Vol21.No1.2018.687.
(5) Kornelius, G.; Forbes, P.; Garland, R. Determination of South African Carbon Dioxide Emission Factors for Selected Solid Fuels. *S. Afr. J. Sci* **2026**, *122* (3/4). https://doi.org/10.17159/sajs.2026/21059.
(6) US EPA. *GHG Emission Factors Hub*. https://www.epa.gov/climateleadership/ghg-emission-factors-hub (accessed 2026-05-18).
(7) MARCOGAZ. *Liquefied Natural Gas (LNG) Quality Database | Marcogaz*; 2024. https://www.marcogaz.org/publications/liquefied-natural-gas-lng-quality-database/ (accessed 2026-05-18).
(8) The Norwegian Environment Agency, Statistics Norway, Norwegian Institute of Bioeconomy Research. *Greenhouse Gas Emissions 1990-2023: National Inventory Document*; 2025. https://www.miljodirektoratet.no/publikasjoner/2025/mars-2025/greenhouse-gas-emissions-1990-2023--national-inventory-document/ (accessed 2026-05-18).
(9) Environment and Climate Change Canada. *Emission Factors and Reference Values*; 2025. https://www.canada.ca/en/environment-climate-change/services/climate-change/pricing-pollution-how-it-will-work/output-based-pricing-system/federal-greenhouse-gas-offset-system/emission-factors-reference-values.html (accessed 2026-05-18).
